# Should Nurses Smoke?

**Published:** 1921-03-26

**Authors:** 


*To the Editor of* The Hospital.

### Should Nurses Smoke?

*To the Editor of* The Hospital.

Sir,—Undoubtedly a large number of nurses enjoy smoking. It seems to me, therefore, that it is a point to consider that if permission (under proper safeguards) be refused, they will smoke surreptitiously.

It may well be, as "Ierne" suggests, that boys commence to smoke under the impression that it is "manly." I smoke because it is a pleasure, and I cannot logically deny the same pleasure to adult women.

Nurses do not get much leisure, so that the opportunities for excessive indulgence are limited. I hope that my Committee will, one day, authorise smoking in the nurses' and sisters' sitting-rooms (or in special smoking-rooms). It would (on account of fire risk) still be prohibited elsewhere.—Yours truly,

Secretary.

